# Controlled placement of multiple CNS cell populations to create complex neuronal cultures

**DOI:** 10.1371/journal.pone.0188146

**Published:** 2017-11-21

**Authors:** D. Soscia, A. Belle, N. Fischer, H. Enright, A. Sales, J. Osburn, W. Benett, E. Mukerjee, K. Kulp, S. Pannu, E. Wheeler

**Affiliations:** 1 Engineering Directorate, Lawrence Livermore National Laboratory, Livermore, California, United States of America; 2 Physical and Life Science Directorate, Lawrence Livermore National Laboratory, Livermore, California, United States of America; SUNY Downstate MC, UNITED STATES

## Abstract

*In vitro* brain-on-a-chip platforms hold promise in many areas including: drug discovery, evaluating effects of toxicants and pathogens, and disease modelling. A more accurate recapitulation of the intricate organization of the brain *in vivo* may require a complex *in vitro* system including organization of multiple neuronal cell types in an anatomically-relevant manner. Most approaches for compartmentalizing or segregating multiple cell types on microfabricated substrates use either permanent physical surface features or chemical surface functionalization. This study describes a removable insert that successfully deposits neurons from different brain areas onto discrete regions of a microelectrode array (MEA) surface, achieving a separation distance of 100 μm. The regional seeding area on the substrate is significantly smaller than current platforms using comparable placement methods. The non-permanent barrier between cell populations allows the cells to remain localized and attach to the substrate while the insert is in place and interact with neighboring regions after removal. The insert was used to simultaneously seed primary rodent hippocampal and cortical neurons onto MEAs. These cells retained their morphology, viability, and function after seeding through the cell insert through 28 days *in vitro* (DIV). Co-cultures of the two neuron types developed processes and formed integrated networks between the different MEA regions. Electrophysiological data demonstrated characteristic bursting features and waveform shapes that were consistent for each neuron type in both mono- and co-culture. Additionally, hippocampal cells co-cultured with cortical neurons showed an increase in within-burst firing rate (p = 0.013) and percent spikes in bursts (p = 0.002), changes that imply communication exists between the two cell types in co-culture. The cell seeding insert described in this work is a simple but effective method of separating distinct neuronal populations on microfabricated devices, and offers a unique approach to developing the types of complex *in vitro* cellular environments required for anatomically-relevant brain-on-a-chip devices.

## Introduction

*In vitro* microfabricated devices supporting organ-level cell or tissue constructs have gained traction in recent years due to their potential to humanely and effectively meet the increasing demand for low-cost, reproducible, and rapid ways to investigate disease mechanisms, evaluate the efficacy and safety of new pharmaceutical compounds, and assess toxic compounds in more human-relevant models. Known commonly as organ-on-a-chip systems, these platforms could reduce the need for preclinical animal testing in the future by recapitulating the microenvironment of human tissue or organ systems [1–10].

Platforms aimed at developing an *in vitro* central nervous system (CNS) model, or a “brain-on-a-chip,” often contain integrated sensing capabilities, such as microelectrode arrays (MEAs), to measure the electrophysiology of neurons [11–13]. An especially multifaceted *in vitro* system may be required to capture the true functionality of the complex human brain, which includes many distinct, but interconnected regions of neurons and other supporting cells. Yet, most *in vitro* CNS platforms for recording neuronal activity have focused on populations of a single cell type per device [14–22]. A more complex and controlled CNS platform with organizational relevance to the human brain *in vivo* holds promise in moving toward a brain-on-a-chip platform.

Many groups have reported methods of co-culturing multiple cell types into specific regions of a device to study intercellular mechanisms like communication, migration, and intrusion [23–27]. These platforms often rely on permanent physical barriers to separate two or more populations of cells. The barriers contain channels that allow signalling, trophic effects, and, in the case of nervous system cells, connections via processes while maintaining cell body separation [28, 29]. Alternatively, chemically patterning of the platforms’ surface can be used to create distinct, localized regions of the device where cells preferentially bind [11, 30–32]. However, physical or chemical restraints have the potential to alter cellular communication, limiting the ability of these platforms to mimic *in vivo* communication and thus reducing their relevance for studying interactions between cell populations [33, 34]. To address these issues, cellular deposition techniques that do not require permanent physical or chemical surface modifications have also been reported. One method for precisely depositing a single cell type is lithographically patterned stencilling [35]. Other techniques include dielectrophoresis [36] and removable barriers [12, 37], which are effective but currently limited by the attainable size and geometry of the confined regions.

In this study, we have developed a novel, removable insert that enables placement of multiple, distinct cell populations onto an MEA-containing substrate without the use of permanent physical or chemical barriers. The insert is able to seed cells into smaller regions compared to other published removable barrier systems (<1.2 mm diameter versus >5 mm in other studies), and in closer proximity to neighboring regions (100 μm vs. 500 μm in other studies) [12, 37]. This is the first technology that enables microscale patterning of neighboring cell populations, each with different compositions and cellular densities; a capability that will be key as the organ-on-a-chip community continues to strive towards more complex systems. Due to its unique tapered path geometry, it is also the most rapid and straightforward way to direct cells from a standard micropipette to a small, defined region on an MEA. Lastly, the insert is highly customizable, allowing placement of cells into regions of many sizes and geometries while still using standard size cell culture wells and micropipettes.

To demonstrate how this approach could create a more anatomically-relevant *in vitro* platform, we show examples of the size and arrangement possibilities allowed by the insert. This study describes the fabrication of two versions of the insert and custom MEA device and demonstrates the insert’s ability to localize primary rodent hippocampal and cortical neurons to specific regions of the MEA surface. Because the insert is removable, it allows neurons to grow uninhibited and establish connections between neighboring regions. We also demonstrate that the health and longevity of the neuronal cultures are not affected by the insert, and that hippocampal and cortical neurons co-cultured over several weeks *in vitro* show distinct bursting behavior in each of the neuronal regions. The creation and characterization of this tool is an essential step towards developing an *in vitro* brain model with multiple, organized neuronal and supporting cell types.

## Materials and methods

### Microelectrode device fabrication

**Fig 1:**
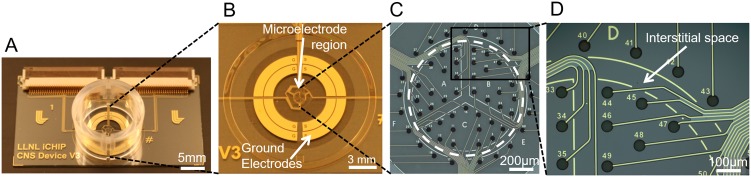
Microelectrode array design. (A) Completed device with well and connectors attached. (B) Cell-containing area of the device. The cell culture well is placed just inside the SU-8 alignment ring. (C) Brightfield micrograph of microelectrode region containing 60 electrodes. Dashed line denotes border between inner and outer region. The inner region is further separated into three subregions, labeled A, B, and C. (D) Electrode detail and interstitial space between two cell placement areas. The metal used for visualization of borders between regions does not intersect any of the electrodes or traces.

The MEA device (Fig 1A) was designed such that a cell culture well could be precisely positioned with respect to the electrodes on the substrate using an alignment ring (Fig 1B, outer-most circle). The alignment ring was lithographically patterned during microfabrication in SU-8 photoresist (Microchem Corp., Westborough, MA). This allows a custom injection-molded polystyrene well (3D Systems, San Carlos, CA) to lock into the ~150 μm tall ring before adhering to the device surface using biocompatible epoxy (Epoxy Technology, Billerica, MA). The MEA was microfabricated as previously described [38], though evaporated gold was used in place of sputtered platinum for the trace metal. Briefly, four-inch SiO_2_ wafers (University Wafer, South Boston, MA) were patterned with gold electrodes/traces and subsequently, polyimide was patterned as an insulator for the traces.

After microfabrication of the arrays, platinum was electroplated onto the exposed gold electrode surfaces to decrease impedance and enhance the signal-to-noise ratio during electrophysiological recordings. Electrodeposition included 80 cyclic voltammograms from -100 mV to +100 mV (vs. Ag/AgCl) at a scan rate of 25 mV s^−1^ in an acidic hexachloroplatinate IV hexahydrate solution. Impedance measurements were taken prior to seeding, as previously reported [38], and measurements ranged from ~50–250 kΩ at 1 kHz.

This cell study focused on separating two populations of neurons: cortical and hippocampal. For this reason, the MEA was divided spatially into two distinct regions, named the inner and outer region (Fig 1C), where the areas of the regions were based on the relative size and orientation of the hippocampus and cortex in the human brain, respectively [39, 40]. Accordingly, the MEA was arranged such that the inner region had had a diameter of ~1.2 mm and a surface area of ~1.13 mm^2^, while the outer region had an area of 142 mm^2^ for the cortical neurons to fully surround the hippocampal region that is just 1% of its size. Throughout these regions there are 62 total electrodes– 60 recording electrodes and two individual ground or reference electrodes. For visualization purposes, as well as to designate the 100 μm wide interstitial space between regions, metal lines (not intersecting any electrodes or traces) were patterned on the substrate (Fig 1C and 1D). Additionally, the inner region was further separated into three smaller subregions providing the capability for increasing the number of unique regions of the device within the same substrate area.

### Fabrication of the cell seeding inserts

**Fig 2:**
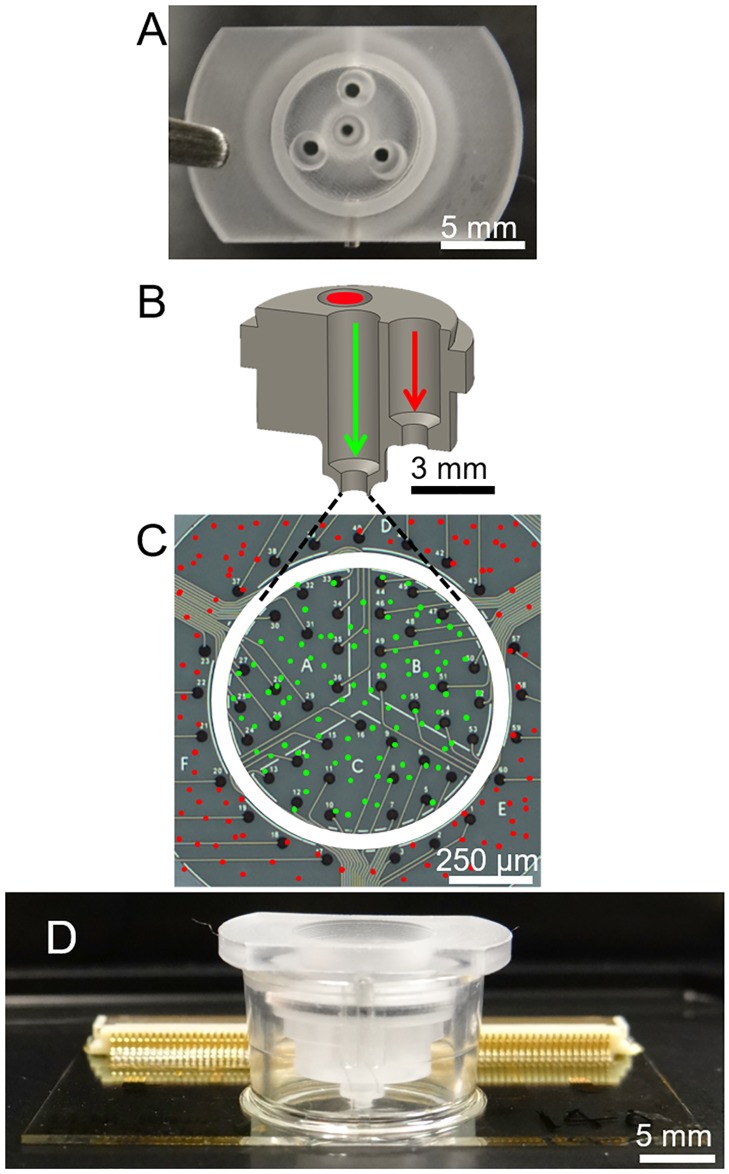
Design and placement of two-cell type seeding insert. (A) Top-down image of the cell-seeding insert, showing four openings into which cells can be pipetted. The three outer openings all lead to a single outer region. (B) Cross section of the lower part, or main body, of the cell-seeding insert. Paths of cells destined for the inner region (green) outer regions (red) are marked by arrows and circle. (C) MEA with a cartoon overlay of where the insert contacts the substrate (white circle), and where cells seeded through the inner (green) and outer (red) insert openings are placed. (D) Device with insert assembly, including insert cap, seated into well. The bottom of the insert is in contact with the MEA substrate.

Two versions of the cell seeding insert were developed to match the geometry of the MEA region of the device. The first, called the two-cell insert, was used to seed two distinct neuronal populations in the inner and outer regions of the MEA with a 100 μm removable barrier along the interstitial space of the substrate during cell seeding (Fig 2). Two individual parts were machined out of polycarbonate using a computer numerical controlled (CNC) lathe then adhered to one another with Epo-tek 301–2 biocompatible epoxy (Epoxy Technology, Billerica, MA).

The top surface of the two-cell insert body contains four 2 mm diameter openings (Fig 2A). The openings allow cells to be easily micropipetted into the insert and settle on the device substrate via gravity. While all three of the outer openings lead to the outer region, only the center opening leads to the inner region (Fig 2A–2C). The center opening tapers near the bottom to an inner diameter of 1.2 mm to match the diameter of the inner region on the substrate (Fig 2C). Since the three outer openings do not contact the MEA surface, all three outer openings deposit cells into the same outer region of the MEA. The removable insert is designed such that a friction fit is created between the inner edge of the cell culture well and the edge of the cap (Fig 2D). Normal movement of the device does not cause displacement of the insert along the plane of the substrate, which would damage or detach the cells, or result in cells leaking between regions. Although tightly fitting, the insert can be removed without disturbing attached cells.

**Fig 3:**
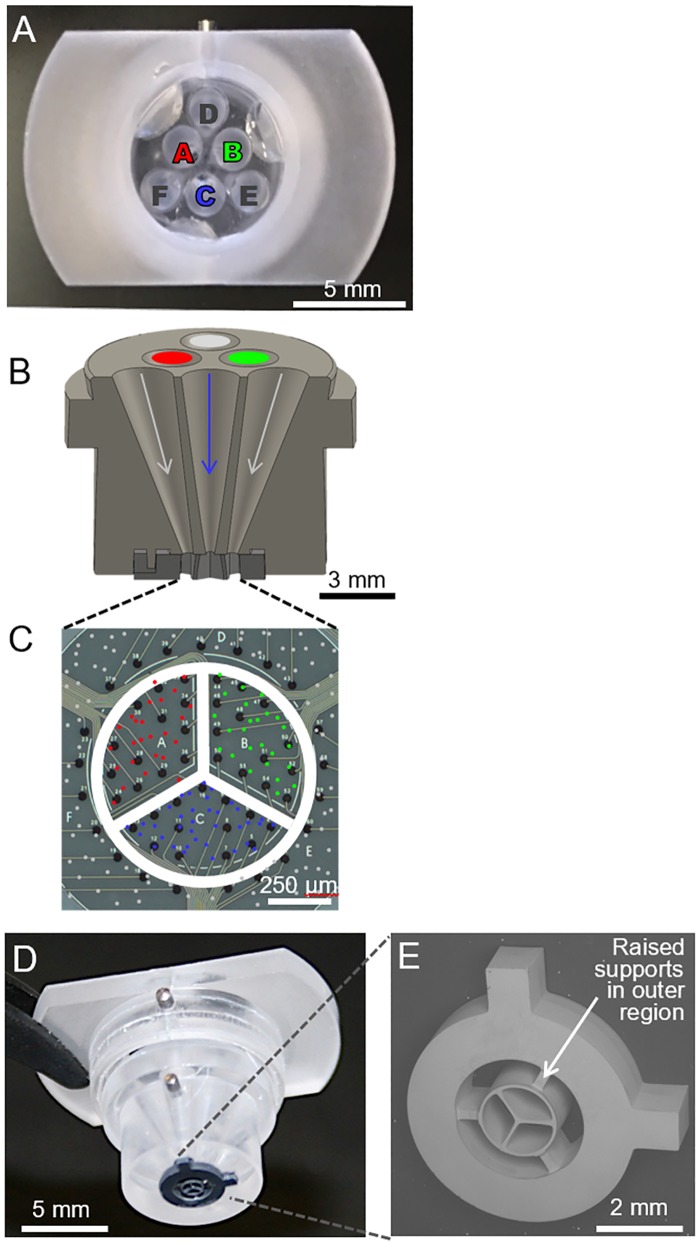
Design of four-cell type seeding insert. (A) Top-down image of the insert, showing six openings into which cells can be pipetted. The three outer openings (D, E, and F) all lead to a single outer region. (B) Cross section of main body of insert. Pathways used to deposit cells for the three inner subregions are labeled by colored arrows while pathways for outer region are in grey. (C) MEA with a cartoon overlay of where the insert makes contact with the substrate (white circle & lines) and where cells seeded through the three inner (green, red and blue) and three outer (grey) insert openings are placed. (D) Assembled insert with microfabricated silicon cell separator. (E) Scanning electron micrograph of microfabricated cell separator. The insert allows three inner subregion cell types to be seeded, along with cells in one outer region. Support structures in outer region do not contact the surface so to allow cells seeded into this any of the three outer openings to attach anywhere within this region.

Similar to the two-cell insert, a four-cell insert was also created by coupling two polycarbonate parts using Epo-tek 301–2. The top surface of the four-cell insert body contains six 2 mm diameter openings in the shape of a triangle (Fig 3A). The openings for cell seeding were made using a conical drill bit to achieve angled orientations and tapered sidewalls throughout (Fig 2B). The three openings at the vertices of the triangle all lead to the outer region of the device, like in the two-cell funnel. However, in the four-cell funnel, the three inner openings lead to individual subregions within the inner region of the device (Fig 3B and 3C). All six openings align to a microfabricated silicon component that provides increased spatial resolution of the three interior subregions of the MEA (Fig 3D and 3E). The silicon divider, which makes direct contact with the substrate, was made by a front- and backside deep reactive ion etching (DRIE) process through a 1 mm silicon wafer. After release from the wafer, the divider was adhered the bottom of the polycarbonate insert using Epo-tek 301–2. Lastly, a thin layer of Epotek 301–2 was applied to the bottom surface of the silicon divider and cured to allow a better seal between the insert and the substrate during cell seeding, and to eliminate the possibility of damage to the electrode surface from the silicon.

### Cell seeding

Prior to seeding, devices were coated with 0.1 mg/mL poly-D-lysine, rinsed four times with sterile DI water and air-dried overnight. The inserts were submerged in 70% ethanol for 30 minutes, rinsed three times in sterile DI water, and air-dried overnight. Immediately prior to seeding, the bottom surface of the seeding insert was dipped into 70% ethanol for 10 seconds to wet the surface and minimize air bubbles. It was then dipped serially into three separate baths of sterile DI water, followed by cell culture media. The surface of the MEA was wetted with 50 μL of cell media immediately prior to placement of the insert in the well.

For seeding using the two-cell insert, primary embryonic rat hippocampal and cortical neurons were purchased from Lonza (Walkersville, MD). Cells were thawed according to the supplier’s instructions and slowly diluted to 1,000 cells per μL in media. For mono-cultured devices, 180,000 cells were seeded, with a total media volume of 350 μL. For co-cultured devices, 180,000 cortical cells were seeded in the outer region, and 30,000 hippocampal cells were seeded into the inner region. After 24 hours, inserts were removed, and cell media was exchanged. Gas-permeable caps, made from a custom polytetrafluoroethylene (PTFE) ring and a fluorinated ethylene-propylene (FEP) membrane (ALA Scientific, Farmingdale, NY), were then placed over the cell culture wells to maintain sterility and allow for gas exchange. Cultures were maintained with bi-weekly 50% media exchanges. Media consisted of Primary Neuron Basal Medium (PNBM) supplemented with 2mM L-glutamine, 50 μg/mL gentamicin, 37 ng/mL amphotericin, and 2% NSF-1. Cells were maintained in a humidified incubator (5% CO_2_, 37°C).

### Cell migration analysis

To monitor the position of cells seeded using the two-cell insert, primary hippocampal neurons were fluorescently labeled with either DiO (green) or DiL (red) lipophilic tracer dyes (Invitrogen) using standard protocols prior to seeding. One cell type was used for the analysis to avoid confounding variables. Once labeled, the cells were deposited through openings in the insert leading to the inner and outer region of the MEA, respectively. Devices were imaged on DIV 1 (immediately after insert removal), DIV 2, DIV 7, and DIV 22 to record distribution of the fluorescent cells. Images were analyzed using cellSens Dimension (Olympus). Fluorescence intensity was quantified for the inner and outer regions of the electrode array. For each day, fluorescence for the inner and outer regions was calculated as a percentage of total fluorescence (inner and outer regions). Three replicate devices were used for these analyses. Data is represented as mean ± standard deviation. P values were calculated using an unpaired t-test. Data were considered statistically significant for p values < 0.05.

### Immunocytochemistry

Cells were fixed with 4% paraformaldehyde, rinsed three times with phosphate buffered saline (PBS) and permeabilized with cold 100% methanol. Cells were stained with a primary mouse antibody to neuron-specific class III beta-tubulin (Tuj-1, Neuromics, Edina, MN, 1:100 dilution) overnight at 4°C. After primary antibody incubation, cells were rinsed three times with PBS and stained with secondary antibodies for one hour at 37°C. The secondary antibody used was a goat anti-mouse linked to Alexa Fluor 488 (1:100 dilution, Life Technologies, Eugene, OR). After secondary antibody incubation, the cells were rinsed three times with PBS. Nuclei were stained with diamidino-2-phenylindole (DAPI, ThermoFisher, 300 nM) for 20 minutes and then rinsed with PBS before imaging.

### LDH assay

Lactate dehydrogenase activity (LDH) was measured in media at DIV 14 and DIV 28 using an LDH kit (Sigma Aldrich). LDH activity was normalized for each group by seeded cell number for comparison. Data is expressed as the mean ± standard deviation. P values were calculated using a one-way analysis of variance (ANOVA), followed by Tukey’s test for multiple comparisons. Data were considered statistically significant for p values < 0.05.

### Electrophysiology recording and processing

For electrophysiology measurements, devices were placed on a heated stage that permitted connection to the recording system while maintaining a constant temperature (37°C). Electrophysiology measurements were made from each MEA device for three minutes using a multi-channel recording system (AlphaLab SnR, Alpha Omega, Alpharetta, GA). Voltage recordings were sampled at a frequency of 22.3 kHz and bandpass filtered between 268 and 8036 Hz. A spike threshold of -31 μV was set as the lower limit for defining an action potential spike (~2.1 times the baseline noise seen on all channels). Raw spike data was then imported into RStudio software (Boston, MA) for further processing and analysis. The data from each device was processed using a custom code in R to remove noisy electrodes (>4000 spikes during a three-minute recording window), noise artifacts seen across all channels (any event that occurs on all channels within a 100 ms time window), and silent electrodes (<15 spikes during the three-minute recording window). Seven device replicates for each cell type were used for mono-cultured, and six device replicates were used for co-cultured electrophysiology experiments.

### Feature analysis and statistics

For all electrodes, spike and burst features were calculated, and the values assigned to each device were the medians of the feature across all electrodes. Feature analysis was carried out with a custom written R package based off the work of Charlesworth *et al*. [20]. Calculated features included percent spikes in bursts, burst rate, burst duration, firing rate, within-burst firing rate, and coefficient of variation (CV) of interburst interval (IBI). Bursts were defined as having a maximum beginning interspike interval of 0.1 sec, a maximum end interspike interval of 0.2 sec, a minimum interburst interval of 0.5 sec, a minimum burst duration of 0.05 sec, and a minimum number of spikes per burst of 10. These parameters were based on previously published studies [20, 41, 42]. Statistical significance was determined using a Wilcoxon rank-sum test. Peak-trough duration was calculated by isolating single units from multiunit spike data in Offline Sorter^™^ (PLEXON, Dallas, TX). After, sorting of single units, action potential troughs from 90 seconds of single unit spike data were aligned and averaged in Offline Sorter^™^. Statistical differences for peak-trough data was calculated using a Mann-Whitney U test.

## Results and discussion

### Controlled deposition of neurons on MEA surface

**Fig 4:**
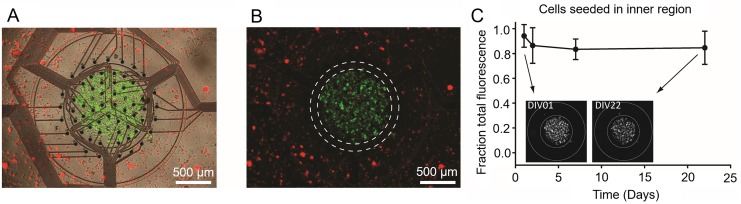
Localization of neurons on MEA device using the two-cell seeding insert. (A) After insert removal, fluorescent images were taken showing localization of fluorescently labeled hippocampal neurons seeded in the inner or outer regions (green and red, respectively). Fluorescent images are overlaid on the phase contrast image to visualize cell seeding relative to electrodes. (B) With overlay removed, localization of both populations can be clearly observed. White circles demarcate the interstitial space between inner and outer region of the MEA surface. (C) Cell movement of hippocampal neurons seeded in the inner region were quantified from DIV 1 to DIV 22, comparing the fraction of fluorescence in the inner region relative to total fluorescence (inner + outer regions, demarcated by white circles in inserts). Data is expressed as the mean ± standard deviation (n = 3).

Differentially labeled hippocampal neurons were used to confirm that the two-cell seeding insert selectively deposited neurons into both regions of the device. Green and red fluorescently labeled neurons were seeded into the inner and outer regions, respectively. Twenty-four hours after seeding, the position of the insert was imaged to determine if movement of the insert had occurred. In all devices observed (n = 35), the insert remained in place. After insert removal and gentle media exchange, the distribution of green fluorescence was assessed. As expected, the majority of green fluorescence (94.1% ± 9.1%) was observed in the inner region of the device (Fig 4). This confirmed that the cell seeding insert made a sufficient seal between the inner and outer regions of the MEA.

Prior to this work, there have been a limited number of published studies involving spatially arranging cells on substrates using removable barriers. Folch *et al*. seeded primary rat hepatocytes on a substrate by depositing through a lithographically patterned PDMS stencil to investigate the feasibility of using a microfabricated removable structure to segregate populations of cells [35]. While they did achieve distinct, attached, patterned regions of cells after removal of the stencil, this method did not allow seeding of different cell types in separate regions. Recently, Garcia-Munoz *et al*. demonstrated localized co-cultures of multiple cell populations using a removable insert. In their study, the insert contained two separate wells (500 μm apart) placed on the surface of a microelectrode array (MEA) [12]. In their device, striatal and cortical neurons were seeded into each well of the insert and electrophysiology testing of mature cells demonstrated connectivity between electrodes in the striatal and cortical regions via a correlation analysis. Das *et al*. reported a similar device for the placement of melanoma cells to study cancer cell migration. This device contained a ring insert for a cell culture dish which effectively separated cells seeded inside the ring from those seeded outside [37]. In the latter two studies, however, the smallest surface area in any of the separated regions was 19.63 mm^2^, with a barrier width of 500 μm. The cell seeding insert developed in this work enables significantly higher resolution patterning; the area of the inner region is 1.13 mm^2^ with a barrier width of 100 μm. These feature sizes allow for seeding of distinct brain regions on the order of the smaller subregions present in the rodent brain [43]. For all experiments with primary neurons in this study, the two-cell insert was used, though cell seeding using the four-cell funnel is demonstrated in the supplemental material and could be implemented in future studies (S1 Fig). It should be noted that the geometry of the openings at the bottom of the insert, particularly for the microfabricated silicon divider of the four-cell insert, are highly-customizable and could be modified to achieve cell deposition regions of many sizes, shapes, and locations.

### Cell localization over time

Since cells seeded through the insert are not constrained by physical or chemical surface features, evaluating cell movement from the original seeding location was of interest. To examine the movement of cell populations once adhered to the MEA surface, devices were seeded with differentially labeled hippocampal neurons in the inner and outer regions of the device using the two-cell insert. The localization and relative movement of the cells was then evaluated during the first 22 days *in vitro*. Quantification of fluorescent intensity over time revealed that neurons seeded in the inner region of the MEA remained localized in the inner region (Fig 4C). There was no significant difference in the percent of total fluorescence in the inner region over the course of the experiment (DIV 1 = 94.1% ± 9.1%; DIV 22 = 84.6% ±13.4%, p > 0.35). Cells seeded in the outer region, however, did exhibit some movement from the outer region (S2 Fig), although this movement was not statistically significant (p > 0.05). In devices without permanent barriers, the lack of permanent features separating cell populations precludes complete control over cell position, especially as cell migration and movement are often key characteristics of certain types of cells. This aspect of our device design provides an interesting model to investigate disease-related cell migration studies in an anatomically-relevant *in vitro* brain platform, such as tumor cell infiltration.

### Assessment of co-culture viability and function

**Fig 5:**
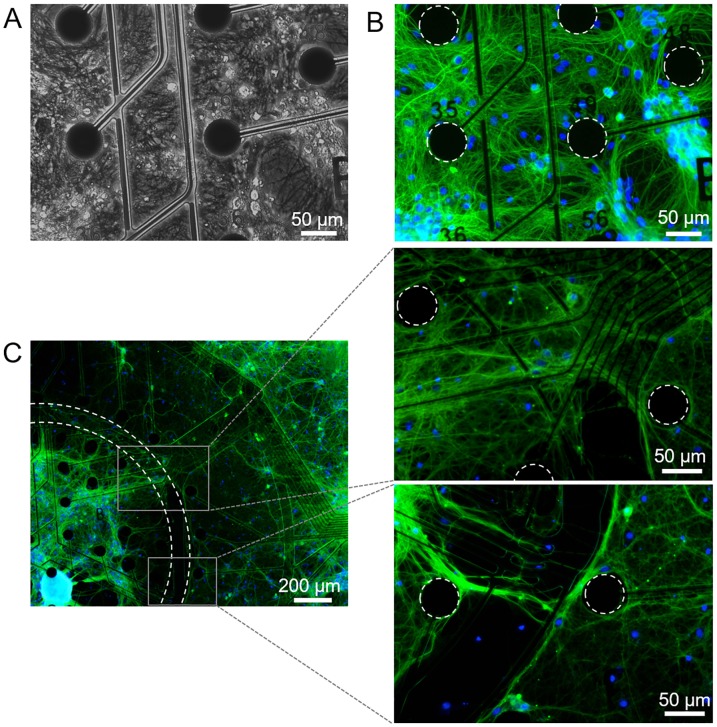
Neuron network development on mono-cultured and co-cultured MEAs. (A) Phase and (B) corresponding fluorescence image of a mono-cultured hippocampal device at DIV 28. A dense, healthy network of processes has formed. Cells were stained with neuron-specific marker Tuj-1 (green) and nuclei marker DAPI (blue). (C) Fluorescence images of a co-cultured device at DIV 28 show processes extending across the interstitial space. The insets show the processes spanning this medial region in greater detail.

To demonstrate the system’s ability to support co-cultured neuronal populations, hippocampal and cortical neurons were seeded into the inner and outer regions of the MEA, respectively. The health and function of these neuronal cultures were assessed through DIV 28 by evaluating both morphology and electrophysiological activity. Mono-cultured control devices were measured in parallel. Cell attachment and network formation were similar for both co-cultured and control devices (Fig 5). To confirm neuronal process formation between regions, cells were stained for neuron specific class III β-tubulin (Tuj-1, Fig 5B and 5C). Cells and neuronal processes spanned the electrodes in each region and across the interstitial space. LDH release was not significantly different between groups (p = 0.054 at DIV 14, p = 0.578 at DIV 28), indicating that co-culturing neurons using the insert did not adversely affect overall cell health over 28 DIV (S3 Fig).

**Fig 6:**
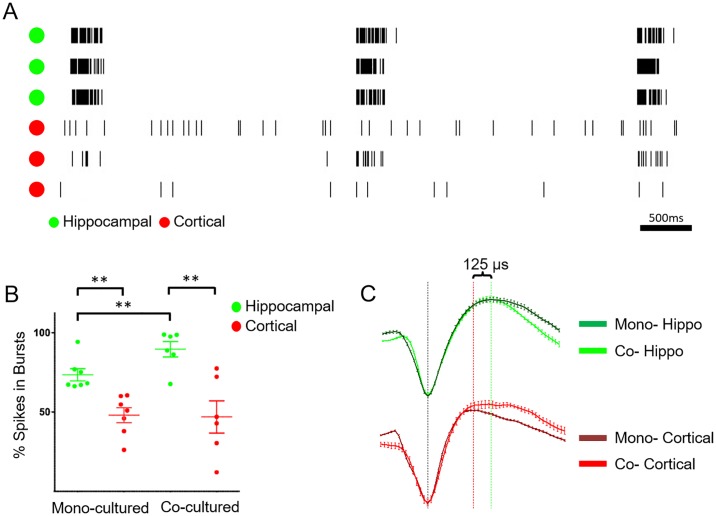
Electrophysiological data recorded from neurons at DIV 28 on MEA. (A) Representative raster plot of action potential events on a co-cultured device. Channels corresponding to electrodes in the inner (hippocampal) region are denoted with green dots, outer (cortical) region channels are labeled with red dots. (B) Comparison of percent spikes in bursts for hippocampal and cortical cells seeded in mono-culture, and from regions of the MEA corresponding to each cell type seeded in co-cultured devices (** indicates p < 0.05). Each data point represents the median value of an entire device. (C) Example hippocampal (green) and cortical (red) waveforms for both mono- (dark) and co- (fluorescent) cultures. Vertical line indicates peak-trough duration times of 324 μs (red) and 450 μs (green) for a mono-cultured cortical and hippocampal cell respectively. Error Bars are ±SEM for 90s worth of action potentials for a given waveform.

Electrophysiology measurements were conducted for both mono- and co-cultured devices to assess functionality after localization with the insert. Co-cultured devices exhibited robust electrophysiological activity for greater than two months *in vitro*. A representative raster plot of spikes measured from a co-cultured device at DIV 28 is shown in Fig 6A. Cells in the hippocampal region (green channels) displayed a distinct bursting pattern, similar to mono-cultured controls. In both mono- and co-cultured cortical neurons (Fig 6A, red channels), cells exhibited less bursting and more single-spike firing activity. These characteristic cell firing patterns agree with studies with similar cell types reported in literature [18, 20, 33, 42].

From these data, several firing features were calculated for mono-and co-cultured devices at DIV 28 including: percent of spikes in bursts, burst rate, burst duration, firing rate, within-burst firing rate, and the CV of the IBI. The percent of spikes in bursts is shown in Fig 6B. Consistent with the qualitative raster plot in Fig 6A and 6B demonstrates quantitatively that differences between the hippocampal and cortical cell spiking patterns are present in both mono- and co-cultured devices.

While percent of spikes in bursts remains distinctly different for the two cell types in co-culture, hippocampal cells in co- or mono-cultures do show distinct changes in spike patterns. Hippocampal cells co-cultured with cortical neurons showed an increase in within-burst firing rate (p = 0.013) and percent spikes in bursts (p = 0.002). These changes that imply communication exists between the two cell types in co-culture. Electrodes from the inner, hippocampal cell region, recorded a significantly higher percent of total spikes in bursts (89.6 ± 4.9%, mean ± SEM) as compared to activity recorded from electrodes within the outer, cortical cell region (47.0 ± 10.1%) (p = 0.013). A similar difference in the percent of spikes in bursts was noted when comparing mono-cultured devices of hippocampal (73.6 ± 3.8%) and cortical cells (48.1 ± 4.7%) (p = 0.002). These data indicate that once seeded with the insert, cells remained localized within their deposited region on the device and displayed characteristic bursting patterns.

Although hippocampal and cortical cells retain this characteristic and expected bursting behavior for each cell type when co-cultured on the device, there are differences when compared to mono-cultures. For example, co-cultured hippocampal cells showed an increase in the percent of spikes in bursts compared to mono-cultured hippocampal cells (p = 0.002). The same trend for the different hippocampal culture conditions is seen when comparing within-burst firing rate, and the slight, though not significant, increase in burst rate and overall firing rate of hippocampal cells (S4 Fig). Communication between the two co-cultured regions may explain these differences and serve as a preliminary indication that co-culturing neurons *in vitro* creates an environment more representative of that seen *in vivo* [44, 45]. Studies have shown that the cortex has excitatory glutamatergic projections to the hippocampus [45] and that the hippocampus serves to integrate and consolidate inputs from many cortical regions into a synchronized signal that can be sent back to the cortical region [44]. This process is essential for memory and learning consolidation [46]. The increased bursting and overall spiking activity seen in the hippocampal region of our co-cultured devices may reflect hippocampal cells integrating glutamatergic cortical signals. A similar effect was reported for modified bursting patterns observed in cultured cells exposed to varying acetylcholine concentrations [18].

To further confirm localization of hippocampal and cortical cell bodies and action potentials to the electrodes in their seeding regions, the action potential waveform characteristics of the two cell types were evaluated (Fig 6C, Table 1). The average peak-trough time for mono-cultured hippocampal and cortical cells were used to evaluate the cell type origins of action potentials seen in each electrode region of co-cultured devices. Mono-cultured cortical cells were shown to have a significantly faster peak-trough time than populations of both mono-cultured hippocampal cells (p<0.05) and co-cultured hippocampal cells (p<0.005). Mitchell *et al*. showed that cortical cells have fast-spiking GABA-ergic interneurons with peak-trough durations of ~200 μs, and excitatory pyramidal neurons that show ~350 μs duration [47]. At 316 μs average duration, this indicates our mono-cultured cortical neurons are representative of the latter cell type. Christian *et*. *al*. demonstrated a similar bimodal trend for hippocampal neurons, with fast (~390 μs) and slower (~630 μs) cells. Hippocampal neurons seeded on both mono- and co-cultured devices in our study exhibited waveform durations similar to the previously reported fast cells with less of a bimodal distribution.

**Table 1:** Summary of waveform peak-trough times for neurons by device type.

Device Type	Avg. waveform peak-trough time^a^	Number of samples
Mono-cultured Hippocampal	445.4 μs ± 36.0 μs	34
Co-cultured Hippocampal	457.2 μs ± 17.4 μs	47
Mono-cultured Cortical	316.5 μs ± 16.2 μs	70
Co-cultured Cortical	570.3 μs ± 55.2 μs	192

^*a*^ Data is given as value ± SEM.

Interestingly, cortical cells demonstrated a significantly slower peak-trough time when co-cultured compared to mono-cultures (p<0.05). This may not be due to physiological changes to the cells themselves but instead may be due to the presence of signals from both cell regions on electrodes near the borders of the two regions. This is further supported by the larger data spread for percent spikes in bursts in the cortical region of the co-cultured devices when compared to the mono-cultured devices (SEM of 10.1% vs. 4.7%). We hypothesize that these two observations are not characteristics of the cells themselves but instead an artifact of the cortical region electrodes also recording the spiking activity of a small percentage of hippocampal processes. This may result from the proximity of the outer region electrodes to the interstitial space and inner region. When action potentials are recorded from processes further from the cell body they show a longer duration waveform [48]. This would be expected for action potentials originating from hippocampal region cells but being recorded on the outer cortical region electrodes. While both the distinctions between and causes behind the mono and co-cultures may be difficult to elucidate and correctly characterize, we believe they are key to understanding both the function and organization of each individual brain region and the entire brain within which these regions reside.

## Conclusions

Two novel cell-seeding inserts were developed for use in an *in vitro* brain-on-a-chip platform. The inserts enable the highest known resolution for patterned deposition of multiple cell types onto an unmodified substrate, allowing uninhibited growth of separate cellular regions. Seeding of multiple cell types onto distinct regions of an *in vitro* platform allows the creation of heterogeneous microenvironments to study complex interactions between neighboring populations of cells, such as tumor migration or propagation of electrophysiological activity between healthy and diseased regions of the brain. Additionally, the insert design and function allows use of standard cell culture methods to focus cell populations of varying composition and density into regions separated by only 100 μm, which has not been demonstrated in previous reports. This high-resolution deposition facilitated by both cell inserts allows for the seeding of anatomically-relevant ratios of cells within very small sub-regions of the array. The two-cell insert was used to spatially seed primary hippocampal and cortical neurons into distinct regions of an MEA and did not adversely affect cell morphology, health, or functionality. Both populations of neurons on co-cultured devices exhibited robust growth and electrophysiological activity over several weeks *in vitro* and demonstrated distinct firing features which in some cases were altered when seeded in co-culture versus mono-culture. This platform provides the ability to achieve a more complex *in vitro* CNS system, including additional brain regions organized with anatomical relevance. A more complex *in vitro* brain model could lead to a greater understanding of organ-wide communication in the brain, development of relevant models for neurological disease or cancer cell migration, and interrogation of chemical agents, pharmaceuticals or therapeutics.

## Supporting information

S1 Fig Demonstration of high resolution cell deposition with four-cell type seeding insert. As a proof of concept for more complex studies and to demonstrate the engineering possibilities of this technology, the four-cell insert was also tested for functionality in seeding multiple cell populations. In this experiment, human cardiac microvascular endothelial (hCMEC/D3) cells stained with lipophilic dyes were used. (A) Cell pathways for the three inner subregions used to deposit hCMEC/D3 cells are labeled by colored arrows. (B) Fluorescence micrograph of three cell populations seeded into the three inner subregions of the device. Each of the three subregions exhibits a ~73% reduction in surface area as compared to the inner region using the two-cell insert (~0.31 mm^2^ vs. ~1.13 mm^2^, respectively). Briefly, for seeding using the four-cell insert, human cerebral microvascular endothelial cells (hCMEC/D3) purchased from Cedarlane Laboratories (Burlington, Canada) were stained using three lipophilic dyes (Vybrant MultiColor Cell-Labelling Kit, Molecular Probes) per manufacturer’s protocol. Cells in suspension (1 x 10^6^ cells/mL) were incubated for 5 minutes at 37°C with cell-labelling solution, spun down at 200 g for 5 minutes and rinsed three times in media before resuspension in warm medium (EndoGRO-MV Complete Media, Millipore) immediately prior to seeding.
(PNG)

S2 Fig Cell movement assessment of cell seeded in outer device region with two-cell insert. Cell movement of hippocampal neurons seeded in the inner region were quantified from DIV1 to DIV22, comparing the fraction of fluorescence in the outer region relative to total fluorescence (inner + outer regions, demarcated by white circles in inserts). Data is expressed as the mean ± standard deviation (n = 3).
(PNG)

S3 Fig Normalized LDH activity across all groups at DIV 14 and 28. Data is expressed as the mean ± standard deviation. For each DIV n = 2.
(PNG)

S4 Fig Burst features calculated from electrophysiology data. Bars represent the mean ± SEM. In comparing hippocampal vs. cortical neurons in both mono- and co-cultured devices, two comparisons showed statistical significance using a Wilcoxon rank sum test. In mono-cultured devices, burst duration (B) was higher in hippocampal neurons than in cortical neurons (p = 0.015). Also in mono-cultured devices, coefficient of variation of the interburst interval (CV of IBI, E) was higher in hippocampal neurons than in cortical neurons (p = 0.03). Lastly, hippocampal neurons on co-cultured devices exhibited higher within-burst firing rate as compared to those in mono-culture (p = 0.02).
(PNG)
