# Supplementary figures and images for: Controlled placement of multiple CNS cell populations to create complex neuronal cultures
Source: PLoS One. 2017 Nov 21;12(11):e0188146. doi: 10.1371/journal.pone.0188146 (PMC5697820; doi:10.1371/journal.pone.0188146)

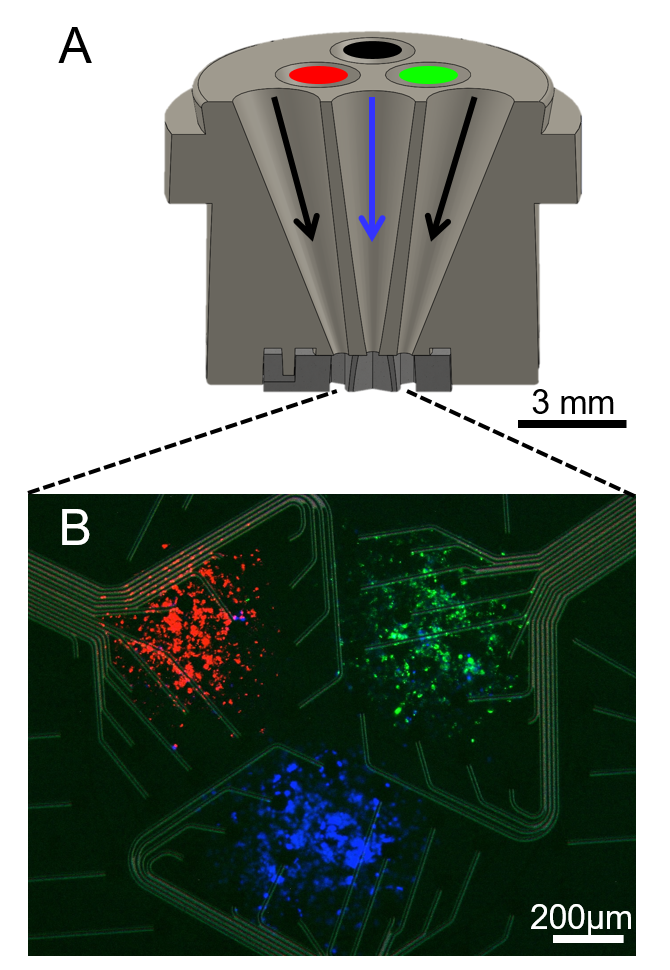

Supplement: S1 Fig — As a proof of concept for more complex studies and to demonstrate the engineering possibilities of this technology, the four-cell insert was also tested for functionality in seeding multiple cell populations. In this experiment, human cardiac microvascular endothelial (hCMEC/D3) cells stained with lipophilic dyes were used. (A) Cell pathways for the three inner subregions used to deposit hCMEC/D3 cells are labeled by colored arrows. (B) Fluorescence micrograph of three cell populations seeded into the three inner subregions of the device. Each of the three subregions exhibits a ~73% reduction in surface area as compared to the inner region using the two-cell insert (~0.31 mm2 vs. ~1.13 mm2, respectively). Briefly, for seeding using the four-cell insert, human cerebral microvascular endothelial cells (hCMEC/D3) purchased from Cedarlane Laboratories (Burlington, Canada) were stained using three lipophilic dyes (Vybrant MultiColor Cell-Labelling Kit, Molecular Probes) per manufacturer’s protocol. Cells in suspension (1 x 10^6^ cells/mL) were incubated for 5 minutes at 37°C with cell-labelling solution, spun down at 200 g for 5 minutes and rinsed three times in media before resuspension in warm medium (EndoGRO-MV Complete Media, Millipore) immediately prior to seeding. (PNG) [file pone.0188146.s001.PNG]

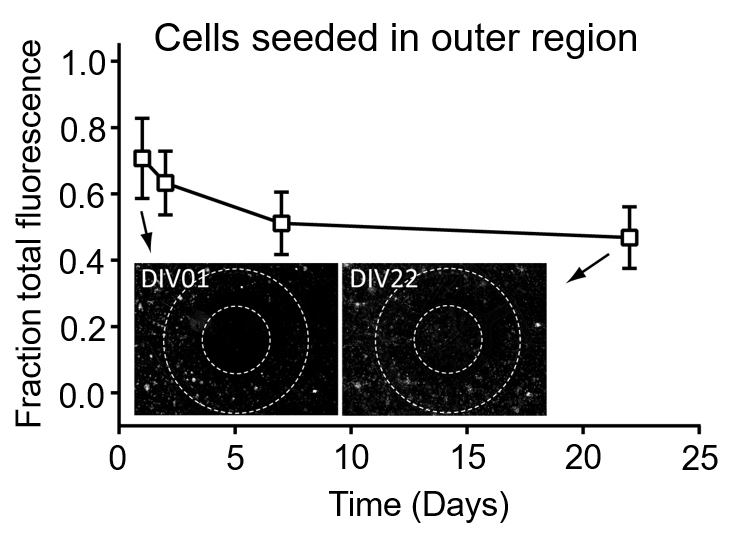

Supplement: S2 Fig — Cell movement of hippocampal neurons seeded in the inner region were quantified from DIV1 to DIV22, comparing the fraction of fluorescence in the outer region relative to total fluorescence (inner + outer regions, demarcated by white circles in inserts). Data is expressed as the mean ± standard deviation (n = 3). (PNG) [file pone.0188146.s002.PNG]

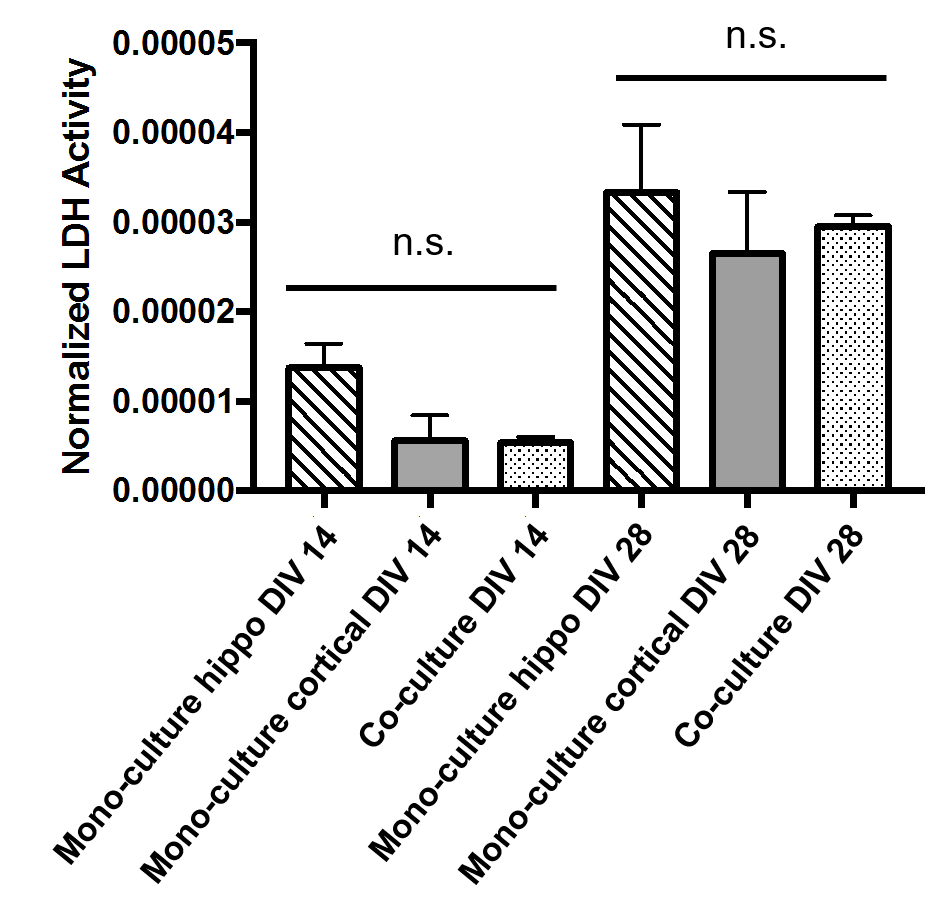

Supplement: S3 Fig — Data is expressed as the mean ± standard deviation. For each DIV n = 2. (PNG) [file pone.0188146.s003.PNG]

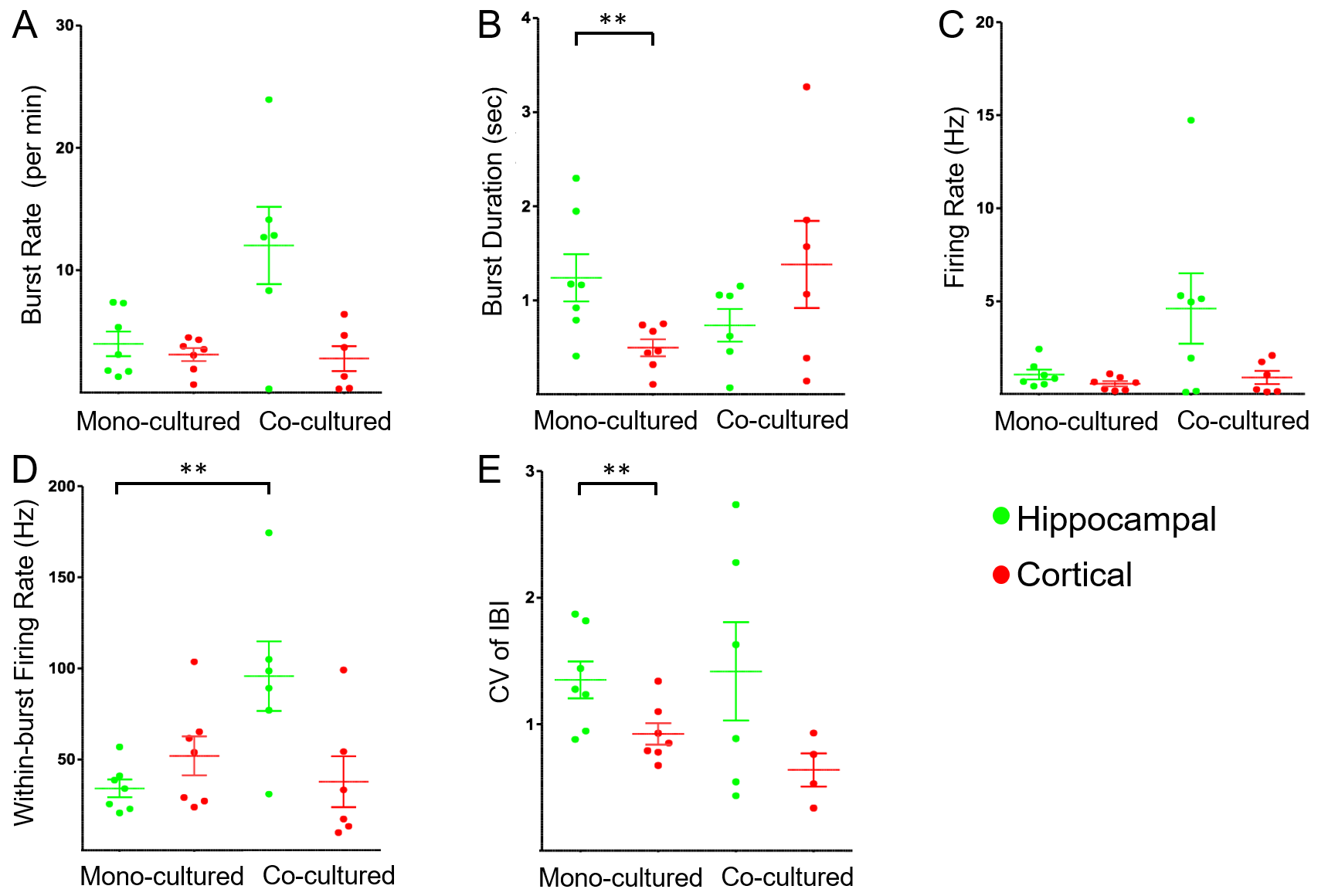

Supplement: S4 Fig — Bars represent the mean ± SEM. In comparing hippocampal vs. cortical neurons in both mono- and co-cultured devices, two comparisons showed statistical significance using a Wilcoxon rank sum test. In mono-cultured devices, burst duration (B) was higher in hippocampal neurons than in cortical neurons (p = 0.015). Also in mono-cultured devices, coefficient of variation of the interburst interval (CV of IBI, E) was higher in hippocampal neurons than in cortical neurons (p = 0.03). Lastly, hippocampal neurons on co-cultured devices exhibited higher within-burst firing rate as compared to those in mono-culture (p = 0.02). (PNG) [file pone.0188146.s004.PNG]
